# Supplementary material for: Distinct myocardial lineages break atrial symmetry during cardiogenesis in zebrafish
Source: eLife. 2018 May 15;7:e32833. doi: 10.7554/eLife.32833 (PMC5953537; doi:10.7554/eLife.32833)
Supplement: Figure 2—source data 1. [file elife-32833-fig2-data1.docx]

**Guerra et al., Distinct myocardial lineages break atrial symmetry during cardiogenesis in zebrafish**

| Gene | Left atria | | | | | Right atria | | | | |
| --- | --- | --- | --- | --- | --- | --- | --- | --- | --- | --- |
|  | CT Sample 1 | CT Sample 2 | CT Sample 3 | CT Sample 4 | CT Sample 5 | CT Sample 1 | CT Sample 2 | CT Sample 3 | CT Sample 4 | CT Sample 5 |
| *Actb* | 17.76 | 18.00 | 17.82 | 18.23 | 17.60 | 17.24 | 17.24 | 17.82 | 18.12 | 18.14 |
| *Pitx2c* | 24.01 | 24.99 | 24.11 | 23.75 | 23.85 | 31.39 | 32.14 | 31.91 | 31.72 | 31.30 |
| *Hsbp6* | 22.02 | 21.91 | 21.77 | 22.32 | 22.03 | 23.47 | 21.98 | 23.10 | 23.39 | 22.96 |
| *Ckmt2* | 16.38 | 17.14 | 16.76 | 17.18 | 17.03 | 17.98 | 18.32 | 18.21 | 18.29 | 18.44 |
| *Scl6a2* | 28.33 | 28.45 | 27.58 | 27.33 | 29.01 | 34.97 | 34.90 | 33.23 | 33.74 | 33.20 |
| *Ptx3* | 28.26 | 28.12 | 28.38 | 29.05 | 28.18 | 27.99 | 27.17 | 28.37 | 28.84 | 29.04 |

**Figure 2 - Source Data 1.** Ct values obtained in RT-qPCR for adult mouse heart (Fig. 2).
