# Supplementary material for: Distinct myocardial lineages break atrial symmetry during cardiogenesis in zebrafish
Source: eLife. 2018 May 15;7:e32833. doi: 10.7554/eLife.32833 (PMC5953537; doi:10.7554/eLife.32833)
Supplement: Figure 7—source data 1. [file elife-32833-fig7-data1.docx]

**Guerra et al., Distinct myocardial lineages break atrial symmetry during cardiogenesis in zebrafish**

| Gene | Atria of *meis2b^-/-^* | | | Atria of *meis2b^+/+^* | | |
| --- | --- | --- | --- | --- | --- | --- |
|  | CT Sample 1 | CT Sample 2 | CT Sample 3 | CT Sample 1 | CT Sample 2 | CT Sample 3 |
| *rpl13a* | 17.10 | 19.31 | 19.24 | 17.31 | 19.38 | 19.10 |
| *pitx2c* | 30.80 | 32.12 | 32.15 | 25.86 | 27.73 | 27.43 |
| *col18a1* | 25.27 | 23.83 | 27.13 | 22.91 | 22.92 | 26.49 |
| *stab2* | 27.48 | 27.43 | 23.66 | 25.53 | 26.91 | 22.28 |

**Figure 7 - Source Data 3.** Ct values obtained in RT-qPCR for adult zebrafish heart (Fig. 7).
