# Supplementary material for: Distinct myocardial lineages break atrial symmetry during cardiogenesis in zebrafish
Source: eLife. 2018 May 15;7:e32833. doi: 10.7554/eLife.32833 (PMC5953537; doi:10.7554/eLife.32833)
Supplement: Supplementary file 1. — Set of primers used to clone in situ hybridization probes. Set of primers employed for RT-qPCR and qPCR analyses. [file elife-32833-supp1.docx]

**Title: Distinct myocardial lineages break atrial symmetry during cardiogenesis in zebrafish**

**Authors:** Almary Guerra^1*^, Raoul F. V. Germano^1,2*^, Oliver Stone^1^, Rima Arnaout^3^, Stefan Guenther^4^, Suchit Ahuja^1^, Verónica Uribe^1^, Benoit Vanhollebeke^2^, Didier Y. R. Stainier^1^, Sven Reischauer^1‡^

**Supplementary Data**

**Supplementary Tables 1-3**

Guerra et al., Supplementary Table 1

|  | Probe | Forward | Reverse | Amplicon size |
| --- | --- | --- | --- | --- |
| a | *meis2b* HRMA primers for F0 | 5’-GCTGCTGGCG  CTGGTGTTTGA-3’ | 5’-GATGAACACA  CATCTCCTCCAG-3’ | 86 bp |
| b | *meis2b* PCR primers for F1 sequencing | 5’-CCTAACCGCA  ACAATAAACCA-3’ | 5’-TTCAGCCAAG  CATCCACATA-3’ | 276 bp |
| c | PCR primers for *meis2b* WT allele | 5’-CCTAACCGCA  ACAATAAACCA-3’ | 5’-CCCGGCTCTC  TCGGTGTGCAT-3’ | 210 bp |
| d | PCR primers for *meis2b^s988^* allele | 5’-CCTAACCGCA  ACAATAAACCA-3’ | 5’-GACTCCCGG  CTCTCTGCG-3’ | 217 bp |
| e | *GAL4FF* primers | 5’-TTTCCTCGTTGGATTAGAT  CGCAGTTTGCTCTAAGCATC  AACAGAAGTGTACCATGAAG  CTACTGTCTTCTATCGAAC-3’ | 5’-TACAACTCAAGTTGTTGA  TCTGTAAACGAGCTTACCC  GTTGAGCCATCAGTCAGAA  GAACTCGTCAAGAAGGCG-3’ | 640 bp |

**Supplementary Table 1.** Set of primers used to genotype *meis2b* mutant and WT alleles, and to generate *TgBAC(meis2b:Gal4FF)^bns15^*.

Guerra et al., Supplementary Table 2

| Probe | Forward | Reverse |
| --- | --- | --- |
| *meis2b* | 5’-CAGATCACAATCCAGCATCC-3’ | 5’-TCTGATGGCTAGGTGAGGAG-3’ |
| *myh6* | 5’-GATTTCCCAACTTACCCGAG-3’ | 5’-TCCATTTCTTCATCCTTCTCAG-3’ |
| *myl7* | 5’-AGACCAACAGCAAAGCAGAC-3’ | 5’-TGATGCTCTACTCATAGTCAAGG-3’ |

**Supplementary Table 2.** Set of primers used to clone *in situ* hybridization probes.

Guerra et al., Supplementary Table 3

| Gene | Forward | Reverse | |
| --- | --- | --- | --- |
| Zebrafish primers | | |  |
| *col18a1* | 5’-GGTGTGAACGGATATAAAGGAG-3’ | 5’-CTTCATATCTGCCAAATCTGTCC-3’ | |
| *pitx2c* | 5’-CACACGGTTTCAGACACCTC-3’ | 5’-CTTGAACCACACTCGGACTC-3’ | |
| *rpl13* | 5’-TAAGGACGGAGTGAACAACCA-3’ | 5’-CTTACGTCTGCGGATCTTTCTG-3’ | |
| *stab2* | 5’-TGAATGTCTGCTGAATCCTCCA-3’ | 5’-CTCTTGTTCTCCCACTGGCT-3’ | |
| Mouse primers | | |  |
| *Actb* | 5’-CTCTGGCTCCTAGCACCATGAAGA-3’ | 5’-GTAAAACGCAGCTCAGTAACAGT-3’ | |
| *Ckmt2* | 5’-TGACCACTTTCTGTTTGATAAGCC-3’ | 5’-TCGTTCCACTTCCTTTAGTCCA-3’ | |
| *Hsbp6* | 5’-GTGCTTCAGCTCCTTTACCA-3’ | 5’-CAACCACCTTGACAGAGATTTCC-3’ | |
| *Pitx2c* | 5’-TGAAGTCGCAGAGAAAGATAAGG-3’ | 5’-CCAAAGCCATTCTTGCACAG-3’ | |
| *Ptx3* | 5’-ACTCCTGCCTCACACTATCTC-3’ | 5’-GCATGATGAACAGCTTGTCC-3’ | |
| *Scl6a2* | 5’-GGCTAGATAGTTCAATGGGAGG-3’ | 5’-TCATTGCTGAACCTGTCCAC-3’ | |
| Mouse primers – ChIP-qPCR | | |  |
| *Pitx2 - Primer pair 1* | 5’-GGCATGTAACCAGATCAGTGTC-3’ | 5’-GCTTGCTTACATTAACTGCCGT-3’ | |
| *Pitx2 - Primer pair 2* | 5’-GGCATGTAACCAGATCAGTGTC-3’ | 5’-GCGGAGGGTTTATAAATCAATCAG-3’ | |
| *Hoxa5* | 5’-TTGTTGTCCAGTCGTAAATCCT-3’ | 5’-CTCTTGCCTCCACCCAACTC-3’ | |
| *Myh6* | 5’-AACTCTGGGATGGGCTCTC-3’ | 5’-AGGAGAGTGCCAAACAGGAC-3’ | |

**Supplementary Table 3.** Set of primers employed for RT-qPCR and qPCR analyses.
